# Synchronization analysis of the uterine magnetic activity during contractions

**DOI:** 10.1186/1475-925X-4-55

**Published:** 2005-10-01

**Authors:** Ceon Ramon, Hubert Preissl, Pam Murphy, James D Wilson, Curtis Lowery, Hari Eswaran

**Affiliations:** 1 Department of Electrical Engineering, University of Washington, Seattle, WA, USA; 2 MEG Center, University of Tübingen. Tübingen, Germany; 3 Department of Obstetrics and Gynecology, University of Arkansas for Medical Sciences, Little Rock, Arkansas, USA; 4 Graduate Institute of Technology, University of Arkansas at Little Rock, Little Rock, Arkansas, USA

## Abstract

**Background:**

Our objective was to quantify and compare the extent of synchronization of the spatial-temporal myometrial activity over the human uterus before and during a contraction using transabdominal magnetomyographic (MMG) recordings. Synchronization can be an important indicator for the quantification of uterine contractions.

**Methods:**

The spatialtermporal myometrial activity recordings were performed using a 151-channel noninvasive magnetic sensor system called SARA. This device covers the entire pregnant abdomen and records the magnetic field corresponding to the electrical activity generated in the uterine myometrium. The data was collected at 250 samples/sec and was resampled with 25 samples/sec and then filtered in the band of 0.1–0.2 Hz to study the primary magnetic activity of the uterus related to contractions. The synchronization between a channel pair was computed. It was inferred from a statistical tendency to maintain a nearly constant phase difference over a given period of time even though the analytic phase of each channel may change markedly during that time frame. The analytic phase was computed after taking Hilbert transform of the magnetic field data. The process was applied on the pairs of magnetic field traces (240 sec length) with a stepping window of 20 sec duration which is long enough to cover two cycle of the lowest frequency of interest (0.1 Hz). The analysis was repeated by stepping the window at 10 sec intervals. The spatial patterns of the synchronization indices covering the anterior transabdominal area were computed. For this, regional coil-pairs were used. For a given coil, the coil pairs were constructed with the surrounding six coils. The synchronization indices were computed for each coil pair, averaged over the 21 coil-pairs and then assigned as the synchronization index to that particular coil. This procedure was tested on six pregnant subjects at the gestational age between 29 and 40 weeks admitted to the hospital for contractions. The RMS magnetic field for each coil was also computed.

**Results:**

The results show that the spatial patterns of the synchronization indices change and follow the periodic pattern of the uterine contraction cycle. Spatial patterns of synchronization indices and the RMS magnetic fields show similarities in few window frames and also show large differences in few other windows. For six subjects, the average synchronization indices were: 0.346 ± 0.068 for the quiescent baseline period and 0.545 ± 0.022 at the peak of the contraction.

**Discussion:**

These results show that synchronization indices and their spatial distributions depict uterine contractions and relaxations.

## Background

Phase synchronization analysis of the uterine electrical or magnetic activity could be used as a tool to quantify the uterine contractions and it may also help us to distinguish between true and false labor. The electrical and the associated magnetic activity of the uterus arise from the generation and transmission of the action potentials in the uterine muscle. It is also possible that the depolarization of the uterine muscle tissue is the result of chemical stimulation at the cellular level. These action potentials or tissue level depolarizations occur in groups and the related measured electrical or magnetic activity appears as a burst activity. The frequency of the action potential within a burst, the duration of the burst, and total number of simultaneously active cells are directly related to the frequency, amplitude, and duration of a uterine contraction[1,2]. The electrical activity, also called electromyography (EMG), of the uterus has been recorded earlier with internal and abdominal surface electrodes [3]. Recently the spontaneous magnetic activity, also called magnetomyography (MMG) of the uterus has also been recorded with a 151-channel SQUID biomagnetometer [2] at the University of Arkansas, Little Rock, USA. The magnetic coils were designed to completely accommodate the frontal area of the gravid abdomen and uterus.

The investigation of synchronization is based on the hypothesis that the uterus remains quiescent throughout most of the pregnancy and close to the time of delivery there is an increase in the electrical activity. It is also assumed that during real labor the uterine cells are tightly coupled and activate in a coordinated way. This will give rise to the increased synchronization of the electrical burst activity throughout the uterine wall leading to the uterine contractions. Here, the synchronization between a pair of channels was defined as a statistical tendency to maintain a nearly constant phase difference over a given period of time even though the analytic phase of each channel may change markedly during that time frame [4,5]. From the collected data set one could examine how the phase synchronization changes between the adjacent channels during a contraction cycle. Based on the resolution of the recording device, several channels should pick up this rhythmic activity.

Our preliminary results show for the first time that the spatial and temporal patterns of phase synchronization and averaged magnetic fields change significantly during a contraction cycle. This was investigated in six subjects exhibiting contractions. Subjects had gestational age between 29 and 40 weeks. Synchronization analysis can be used in further studies to determine the propagation of contractions in the uterine wall and also will help in determining the normal or abnormal contractions for clinical work.

## Methods

### Uterine magnetic field data collection

**Figure 1:**
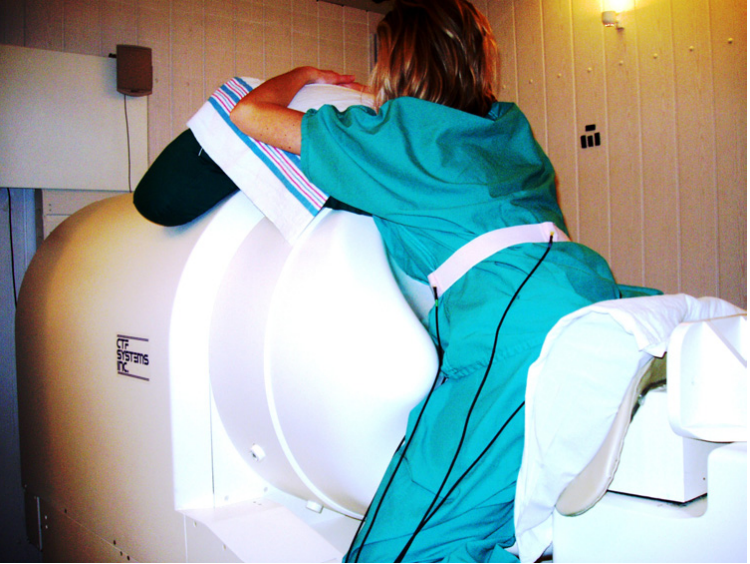
SARA system for measurement of the magnetic activity of the uterine. (Courtesy of CTF Corporation.)

**Figure 2:**
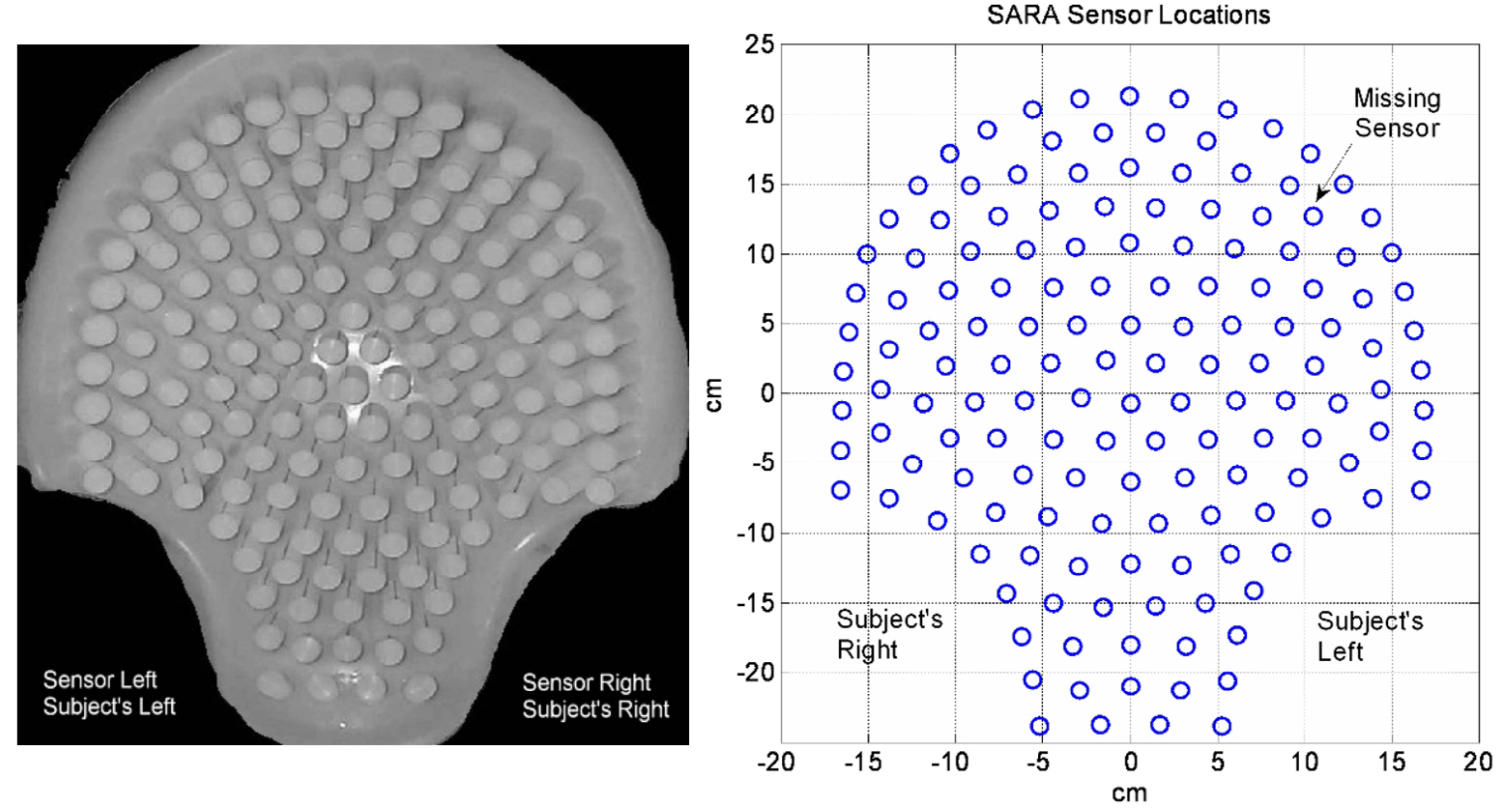
(Left) A frontal photograph of the arrangement of sensor coils. A person sits in front of these coils during data collection. (Right) A schematic layout of the primary sensor coils as seen in front of a subject.

Procedures for collecting the uterine magnetic field data are described elsewhere [1,2]. A brief summary is given here. Transabdominal MMG recordings of six adult female subjects were collected with the 151-channel SARA (VSM MedTech, Ltd., Port Coquitlam, B.C., Canada) system. Here, the acronym SARA stands for SQUID Array for Reproductive Assessment. The sensors are arranged in a concave array that spans the maternal abdomen and the uterus. A photograph of the system during a routine examination is shown in figure 1 and a layout of the sensors is shown in figure 2. A photograph of the sensor coils is in the left side of the figure. A person sits in front of these coils during data collection. A schematic layout of the primary sensor coils is shown in the right side of the figure 2. In this plot, the radiological coordinates are used where right and left are switched. All 151 primary magnetic sensors are spaced approximately 3 cm apart over an area of about 850 cm^2 ^[1]. The primary sensor coils are of 2 cm diameter. The mother simply sits and leans forward against the smooth surface of the array allowing the SQUID (Superconducting Quantum Interference Device) sensors to receive magnetic signals from the entire maternal abdomen [1,2].

The data of six subjects were collected with the above described SARA system. During these recordings, one of the sensor coils was not in operation due to high noise level. The position of this sensor is identified in figure 2. The magnetic signature at the missing sensor is estimated by averaging the data from the surrounding six sensors.

**Figure 3:**
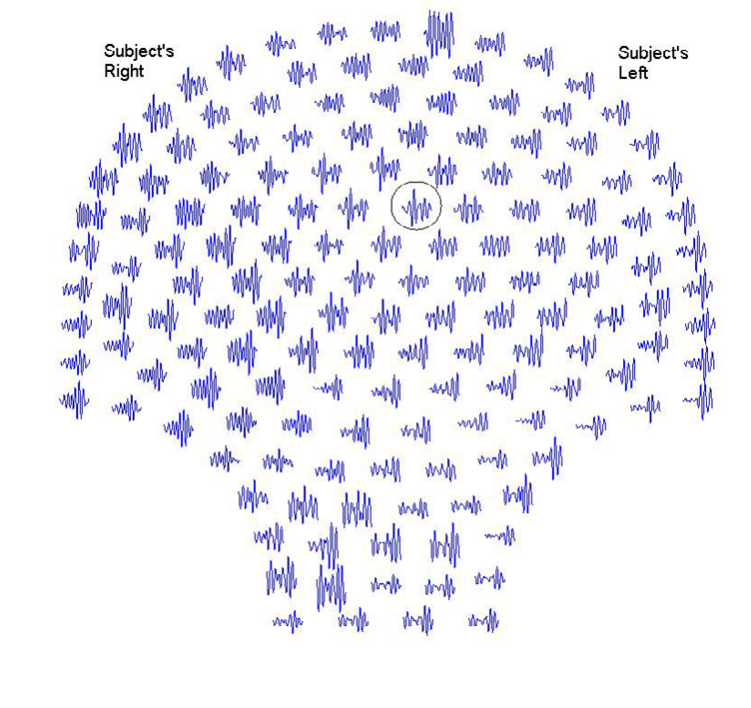
A montage of the 60 sec long uterine magnetic field data.

The subjects recruited in this group, presented themselves at the labor and delivery unit of the hospital complaining of contractions. They were not in active or latent phase of labor and no cervical changes were detected. The study was approved by the Institutional Review Board and a written consent was obtained from each subject. The gestation period of subjects ranged from 29 to 40 weeks. In general, recording sessions ranged from 8 to 12 minutes. For each subject, a continuous 4 minute long data set was selected for synchronization analysis containing at least two contractions. The subject indicated by a finger raise, measured by a light barrier start and end of a perceived contraction. The data was collected with a sampling rate of 250 samples/sec. The data was then downsampled at 25 samples/sec and filtered with a bandpass filter (0.1–0.2 Hz) for further analysis. The bandpass of 0.1–0.2 Hz was selected to extract the signal related to the primary contraction activity of the uterus [1,2]. A montage of 60 second long data set is shown in figure 3. One should note that a typical uterine contraction last 45 to 60 seconds [1] and, generally, MMG activity precedes the perceived contraction.

### Data analysis

The synchronization and decoherence indices between two sensor channels were computed after taking Hilbert transform of the data. The synchronization between a pair of channels was inferred from a statistical tendency to maintain a nearly constant phase difference over a given period of time even though the analytic phase of each channel may change markedly during that time frame [4,5]. The Hilbert transform was applied on the pairs of magnetic field traces (240 second length) with a stepping window of 20 sec duration which is long enough to cover two cycles of the lowest frequency of interest (0.1 Hz). The analysis was repeated by stepping the window at 10 sec intervals.

**Figure 4:**
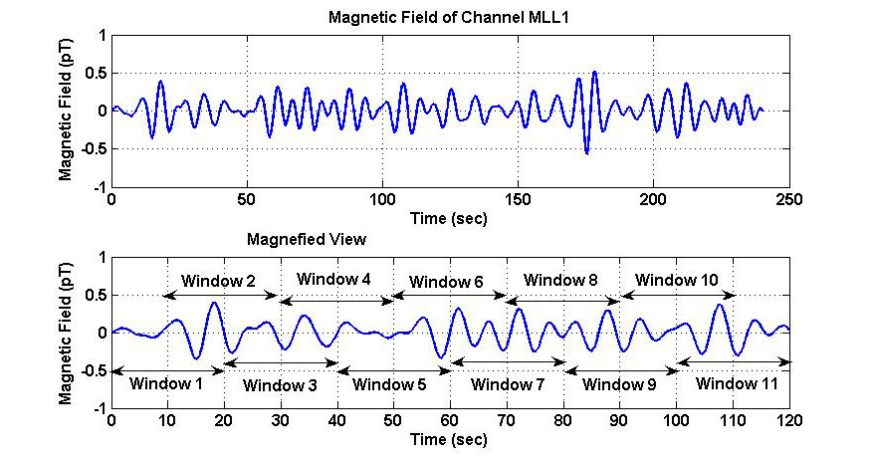
(Top) An example of the magnetic signal in one of the sensing coils, (bottom) magnified view of the signal with 20 second window frames marked on it. The step size for stepping the window is 10 sec. Peaks related to the uterine contractions are very noticeable at 18, 108 and 175 seconds. There is also an irregular contraction activity between 60 to 100 seconds.

A typical magnetic field of a channel and the stepping windows are given figure 4. This channel is identified by a circle in figure 3. The top trace shows the temporal pattern of the magnetic field over 240 sec duration. One can clearly see the peaks of the magnetic activity which are related to contraction peaks. Peak contraction activity is very noticeable near to 18, 108 and 175 seconds. There is also an irregular contraction activity between 60 to 100 seconds. The bottom trace is a magnified view with the 20-seconds stepping windows marked on it.

The phase of the analytic signal shows a sawtooth pattern which is unwrapped to produce a cumulative linear phase of the signal. The phase differences between the two channels were computed by subtracting the phase of one channel from the other. This phase difference was then used to determine the synchronization and decoherence indices. The mathematical techniques for computing synchronization and decoherence indices are given in detail elsewhere [4-6]. We computed synchronization indices based on Shanon entropy function [5]. Phase locking, i.e., synchronization between the phases of two signals within a stepping window was given by Shanon entropy function, *e(t)*, defined as:



where *p*_*i *_was the relative frequency of finding the phase difference modulus of *2π *in the *i*^*th *^bin. The function *e(t) *varied between zero and its maximum value of *e*_*max*_* = ln N*. We used 100 bins (*N = 100*) for the phase difference in a 20 sec stepping window. This phase locking was normalized and called synchronization index, *q(t)*, and is represented as:



The synchronization index, *q(t)*, has a value of zero for uniform distribution of phase differences and a value of one for a spike, or delta, distribution of phase differences between two signals. The decoherence index is defined as the standard deviation of the phase difference between two channels and, in general, varied inversely with the synchronization index [4].

The spatial patterns of the synchronization indices covering the anterior transabdominal area were computed. For this, regional coil-pairs were used. For a given coil, the coil pairs were constructed with the surrounding six coils. This gives us 21 unique combinations of coil pairs to compute the synchronization and decoherence indices for a particular coil. For the coils at the edges, six nearest coils were also used. This provided 21 unique coil pairs for each coil at the edges. The spatial averaging of the synchronization indices was not uniform for the coils at the edges. It was slightly biased towards the inner coils. The spatial averaging of the synchronization indices was uniform for the coils away from the edges.

The synchronization indices were computed for each coil pair. These values were averaged over the 21 coil-pairs and then assigned as the synchronization indices to that particular coil. This analysis was performed for all 151 coils to make 2-D spatial plots of the synchronization indices covering the transabdominal area. The synchronization indices were referenced to a common average reference for plotting and comparative analysis. In the temporal direction, the above procedures were repeated for each 20-sec window.

**Figure 10:**
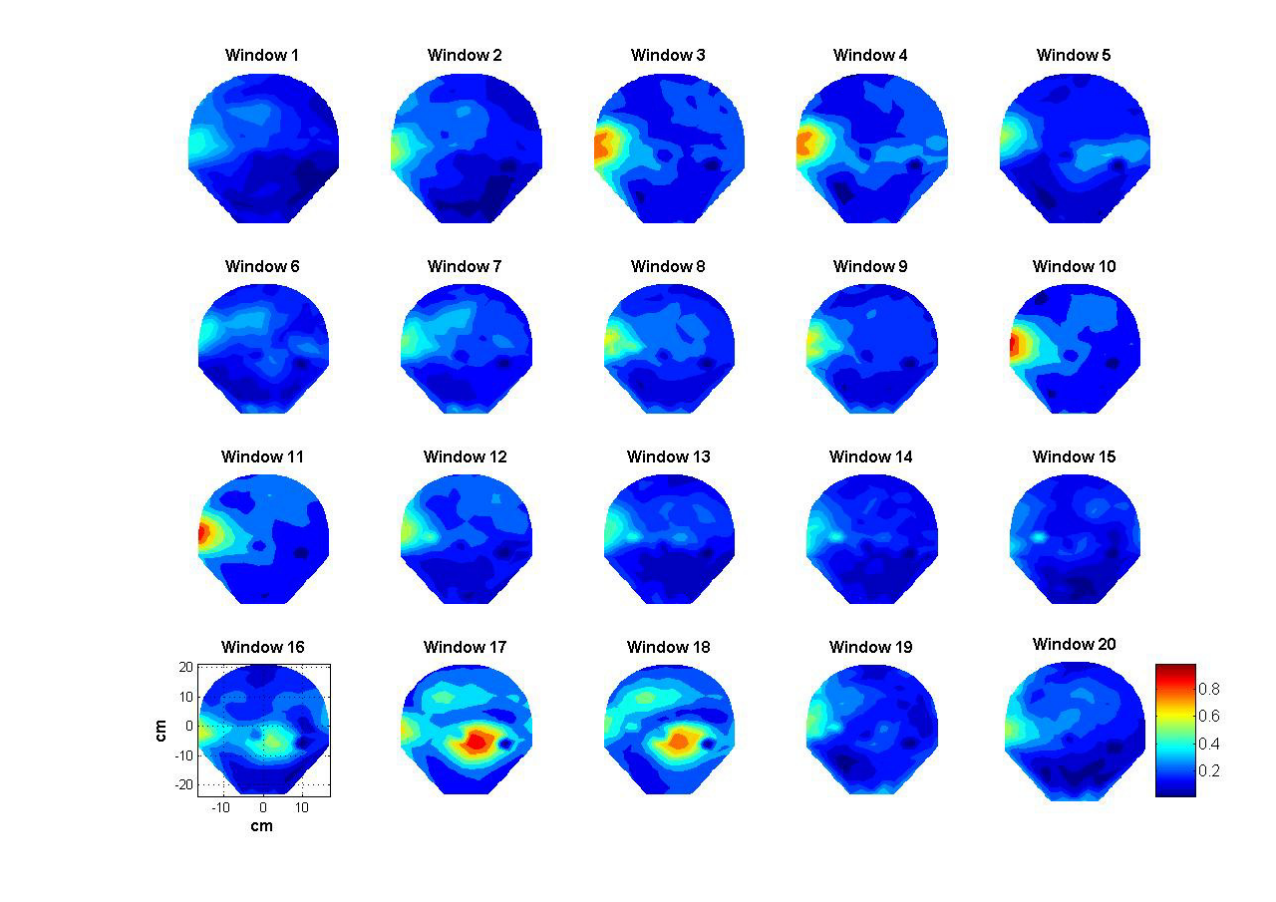
Contour plots of the RMS magnetic field in 20 sec windows.

**Figure 11:**
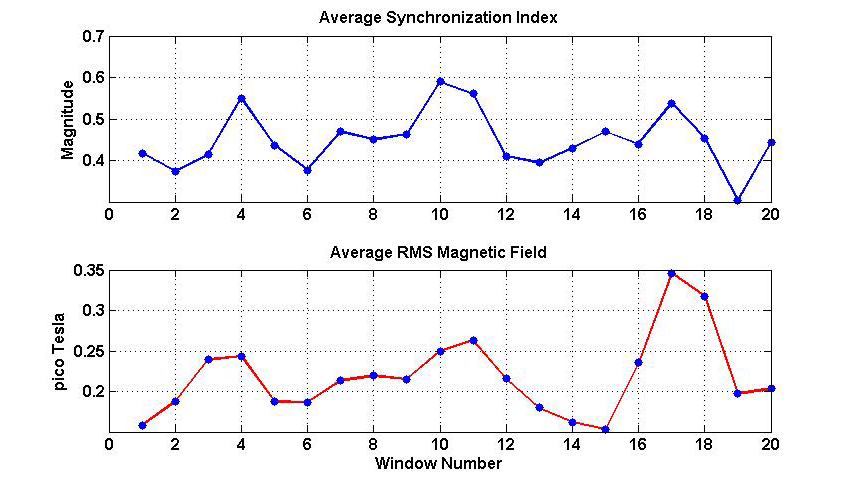
Spatially averaged synchronization indices and the RMS magnetic fields.

The RMS (root mean square) magnetic field for each channel in the 20 sec windows is also computed and presented in figures 10 and 11.

For a comparative analysis, the synchronization and decoherence indices were also computed for the randomly shuffled magnetic field data for each channel. It is anticipated that synchronization indices of the randomly shuffled data will be much lower than the unshuffled data. The random shuffling of the time-series data of a given sensor was done by random permutations. For a coil pair, the random shuffling was independently performed for both sensors. All data analysis was performed using MATLAB 7.1 (The Mathworks, Natick, MA., USA) software on a 2.8 GHz Windows Intel workstation.

## Results

### Two channel synchronization

**Figure 5:**
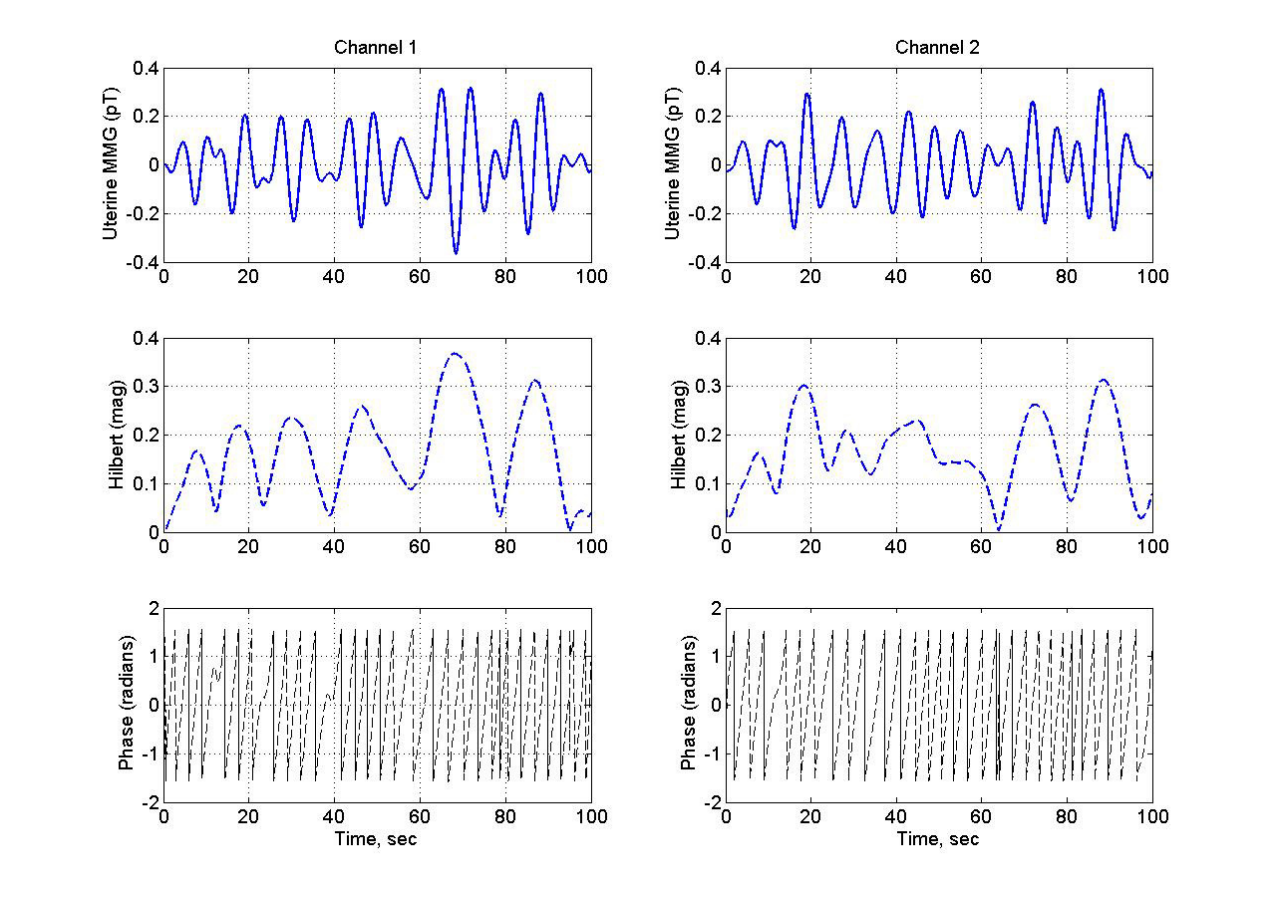
The uterine magnetic fields of two channels in the middle of the transabdomianl area. Also the magnitudes and phases of analytic signal obtained after taking Hilbert transform of the magnetic fields.

**Figure 6:**
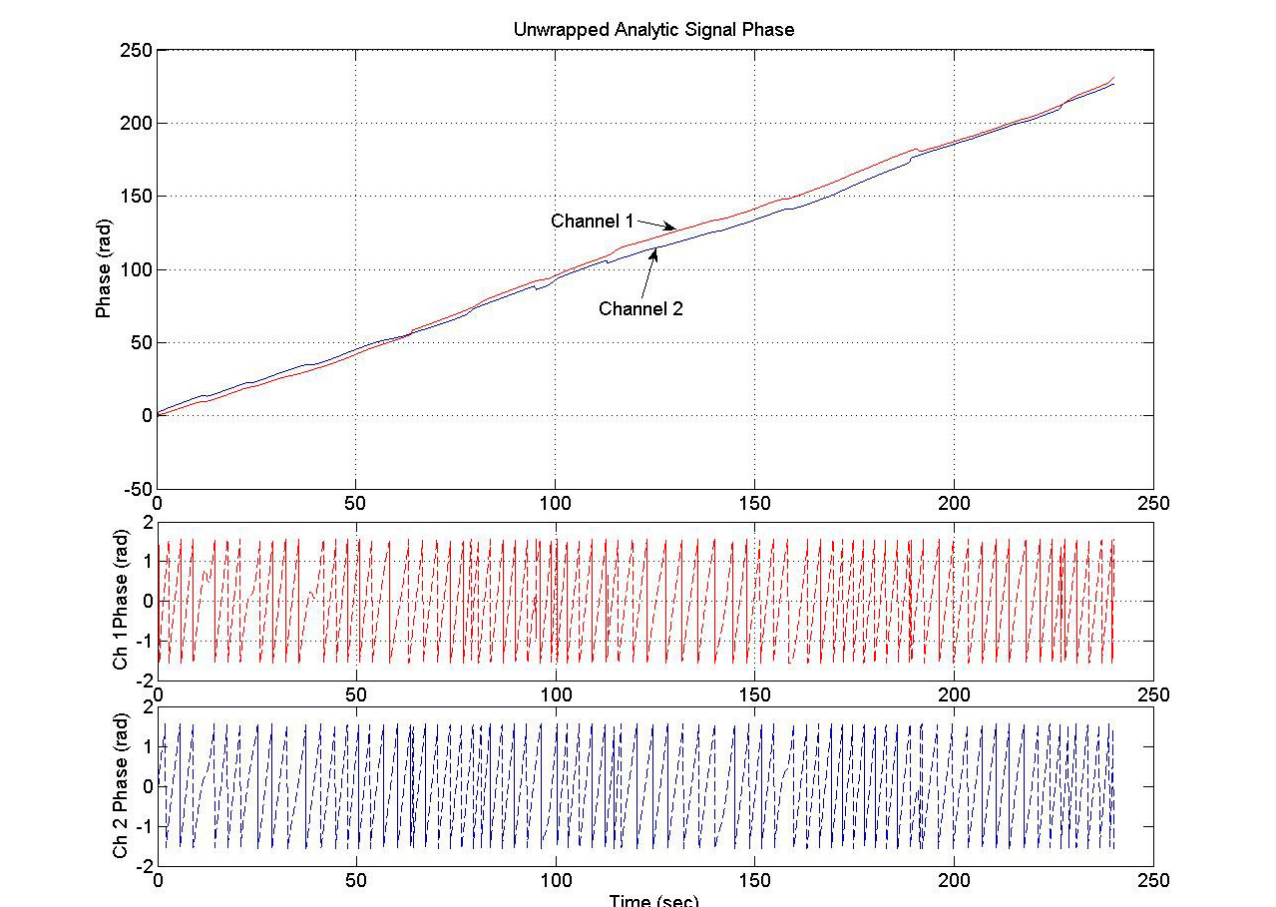
The analytic phases of two signals.

**Figure 7:**
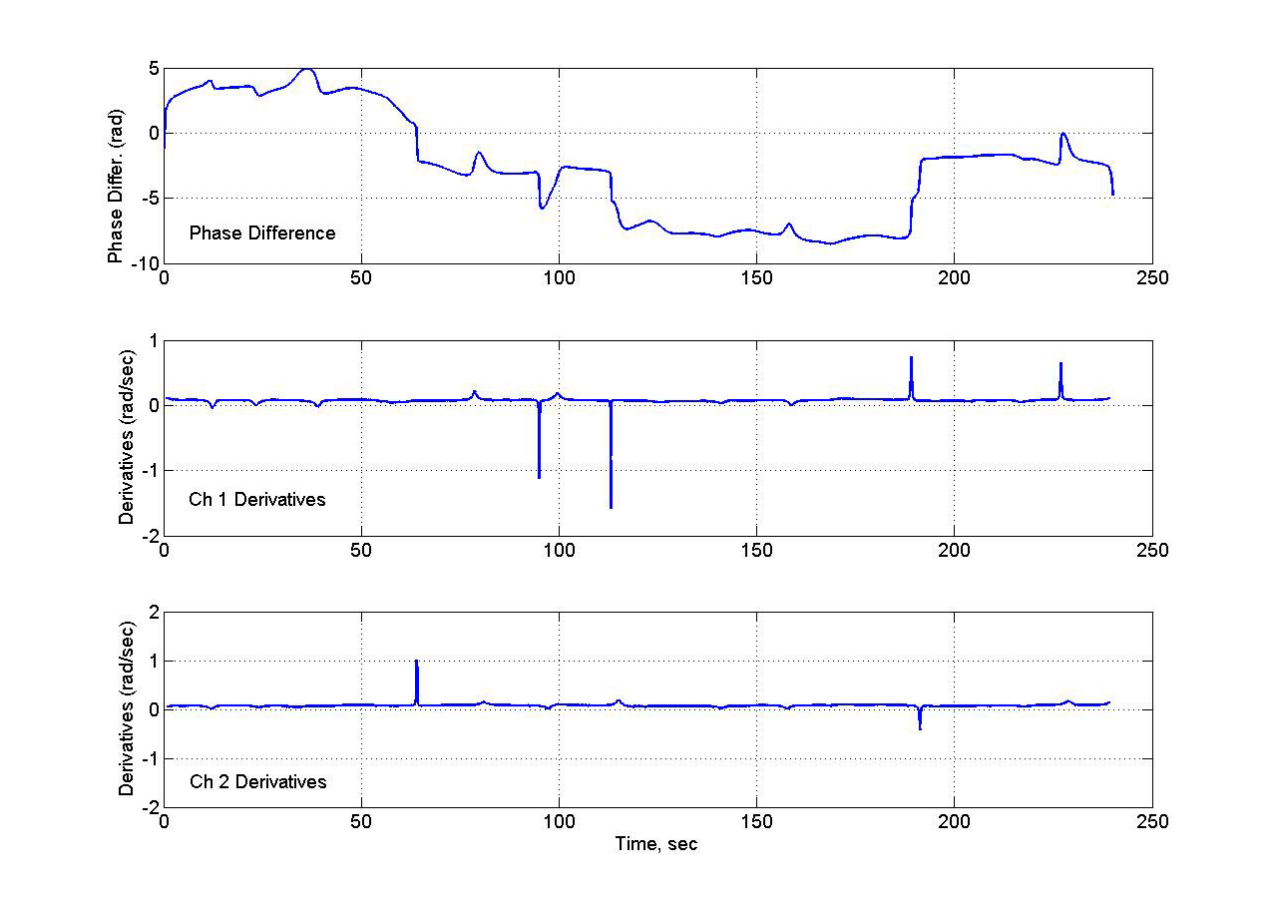
(Top) The phase difference between two signals, (middle) temporal derivative of the analytic phase of one signal, and (bottom) temporal derivative of the analytic phase of the other signal.

**Figure 8:**
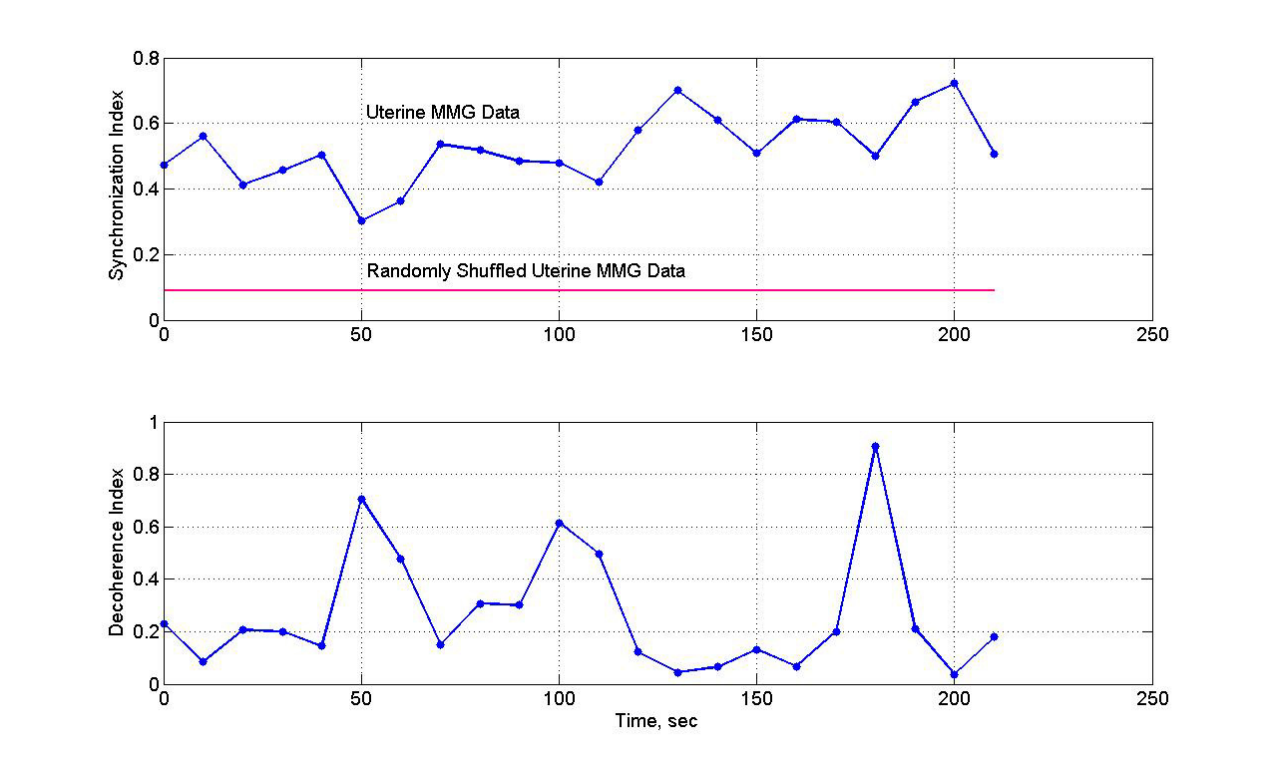
(Top) Synchronization index and (bottom) decoherence index of two signals. The mean value of the synchronization index of the randomly shuffled data is also shown in the top frame.

An example of how the synchronization indices are computed for a pair of channels is given in figures 5, 6, 7, 8. For this we selected two sensor channels in the middle of the transabdominal area which contain moderate level of contraction and relaxation activity of the uterus. The uterine magnetic fields, analytic signal magnitudes and phases are shown in figure 5. For the sake of clarity, this analysis is shown only for the first 100 seconds even though the analysis was performed for the entire 240 sec long data sets. The top row of plots are the uterine magnetic fields of channel 1 and 2. All the left plots are for channel 1 and the right ones are for the channel 2. The middle row of plots are for the magnitude of the analytic signal which was obtained after taking Hilbert transform of the magnetic field data shown in the top row. Similarly, bottom row plots are the unwrapped phases of the analytic signal.

The cumulative analytic phase of the MMG data over a 240 sec period was obtained by unwrapping the phase of the analytic signal, a part of which is shown in figure 5. The analytic phases are plotted in figure 6. Both channels have almost a similar slope of approximately 0.96 rad/sec (or 0.15 Hz) over the 240 sec length of MMG data. The phase difference between two channels is plotted in figure 7, top plot. The middle and the bottom plots are for the phase derivatives (rad/sec) of the channel 1 and channel 2 phases, respectively. Over small sections of time windows (~2–4 sec), it can be observed that phase differences between two channels are constant, i.e., horizontal sections of the phase difference plots. In these short time windows, the two channels are in phase coherence, or synchronized. While in other places the phase difference is noticeably changing signifying that two channels are going in and out of phase. This becomes more visible when one looks at the derivatives of the phases of channel 1 and 2 given in the middle and in the bottom plot. As expected, derivative plots are almost flat lines except for squiggles and some well defined sharp spikes. At the spikes, the changes in the phase differences are very large and one would expect a high decoherence near to those spikes. The calculated synchronization and decoherence indices are shown in figure 8. The synchronization and decoherence indices are almost mirror images of each other. The synchronization indices show the maintained level of synchrony between two signals while the decoherence indices emphasize the episodic departure from the synchrony [4].

### Spatial Synchronization Patterns

**Figure 9:**
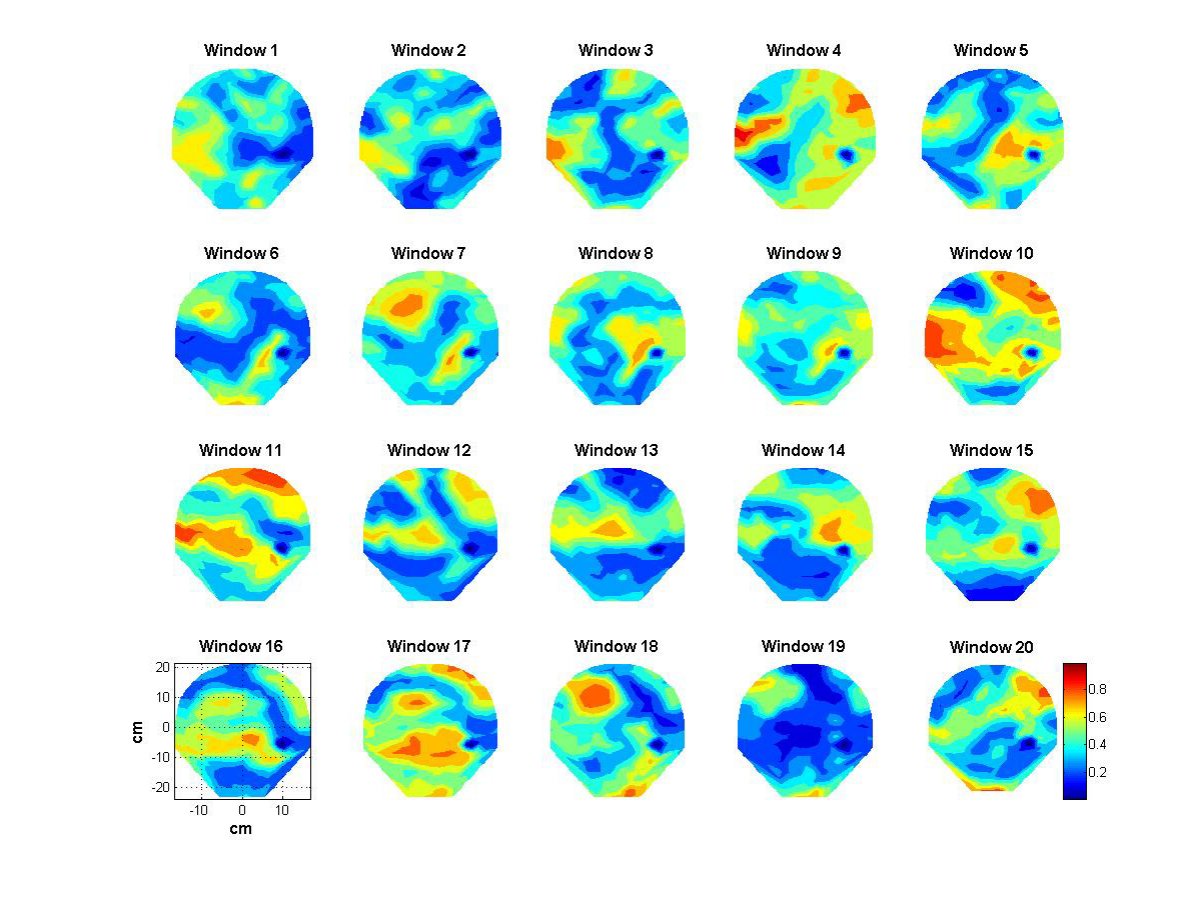
Contour plots of the synchronization indices during 20 sec stepping windows.

Spatial patterns of the synchronization indices and the RMS magnetic fields are given in figures 9 and 10 respectively. The window frames are identified in figure 3. All plots are scaled to the same scale which is given in window 20. The intensity scale is normalized in the range of zero (blue color) to 1.0 (red color). The red areas in the plots depict higher synchronization. Spatial patterns of the synchronization indices show some correlation with the RMS magnetic field patterns. On a window by window visual comparison, the ones that seem to match are 1, 2, 3, 4, 10, 11, 16, 17 and 20, while those that show poor correlation are 5, 6, 7, 8, 9, 12, 13, 14, 15, 18, and 19. Thus, one could conclude that there is some correlation between the synchronization index and the RMS magnetic field. These plots in figure 9 also show that during contraction larger areas of uterine have higher levels of synchronization as compared to the 20 sec window before and after the contraction. For example refer to the plots in windows 9, 10, 11 and 12. Here, windows 10 and 11 are on the contraction peak as shown in figure 4.

The minimum and maximum values of the synchronization indices and the RMS magnetic fields were computed for each window and then averaged over the 20 window values. The average for the minimum values of synchronization indices is 0.3 ± 0.06 and the average for the maximum values is 0.59 ± 0.06. Similarly, for the RMS magnetic fields, the average of the minimum values was 0.153 ± 0.05 *pT *and the average of the maximum values was 0.346 ± 0.03 *pT*. Here the minimum values refer to the windows where the uterine contraction activity is minimum or there is no activity. The maximum values should refer to the windows where there is significant uterine contraction activity.

Spatial averages of synchronization indices and the RMS magnetic fields for each window were computed and are given in figure 11. Both show a similar pattern, i.e., higher averaged values at the contraction peaks as compared to the lower values during baseline activity of the uterus. The range of the average synchronization indices was 0.3 to 0.6. The average synchronization indices of all window frames for the randomly shuffled data were also computed. Its value was 0.088 ± 0.04. Compared to this, the averaged synchronization indices in each frame are much higher (0.3–0.6) for the actual data without random shuffling.

Spatial patterns of the synchronization indices and the RMS magnetic fields of all six subjects were also computed. For each subject we identified the windows where the synchronization activity was high and spread in large areas. These windows were considered to contain large areas of uterine contraction. Similarly, windows were identified where the synchronization activity was low and, in general, relate to the relaxation period of the uterus. In a 4 minute long data set, we identified two window frames for the contraction activity and two for the relaxation activity. The averaged synchronization indices and the averaged RMS magnetic fields for these windows were computed and are given in table 1. The mean values of all six subjects are given at the bottom of the table. Synchronization indices and the RMS magnetic fields, both are higher during contraction period as compared to the relaxation period.

**Table 1:** Average of the of synchronization indices and RMS magnetic fields at the peak of the uterine contraction and at relaxation.

	Synchronization Indices		RMS Magnetic Fields	
	
Subject No.	Contraction	Relaxation	Contraction	Relaxation
1	0.563	0.395	0.305	0.15
2	0.525	0.21	0.190	0.12
3.	0.56	0.35	0.3	0.2
4.	0.56	0.37	0.215	0.15
5.	0.51	0.37	0.28	0.135
6.	0.55	0.38	0.23	0.15
Mean	0.545 ± 0.022	0.346 ± 0.068	0.253 ± 0.048	0.151 ± 0.027

## Discussion

Synchronization indices and their spatial distributions depict uterine contractions. The analysis of the two channel data shows that there are definite changes in the synchronization and decoherence indices over a given contraction cycle of 1–2 minute long data. Based on these observed changes, the spatial patterns of the synchronization changes over the transabdominal cavity were computed. These show that the spatial patterns of the synchronization activity and the RMS magnetic fields change during a contraction cycle. One can follow these changing patterns over two contraction cycles in a 240 sec long data set which we have used.

Here we have restricted our analysis to the primary frequency band of the uterine contraction, viz., 0.1–0.2 Hz which likely represents the plateau and repolarization phase of the action potentials. This is a very low frequency band which forced us to use a stepping window of 20 sec length for computing the synchronization indices. It is a long window which smears the uterine biological information over a 20 sec long window. For a more accurate analysis, closer to the real time-frame, a small window size and a small step size is needed. This would require that the uterine magnetic field data in a higher band of 0.3–1 Hz be used for the phase synchronization analysis. This would provide additional information to what we have reported here. If one wants to study the spikelike activity of the initial inward currents of the tissue, probably a higher frequency band (10–100 Hz) will be more appropriate for data analysis.

Spatial patterns shown in figures 9 and 10 do show similarities in few window frames and also show large differences in few other window frames. One would expect a significant correlation between each frame of synchronization indices of figure 9 with that of the RMS magnetic field shown in figure 10. However, there are reasons for these observed differences. The synchronization indices are averaged over 21 regional coil pairs while the RMS magnetic field is not averaged over the nearby coils. It is computed individually for each coil. This could create differences in the spatial patterns as observed in figure 9 and 10. At the peak of the uterine contraction, one would expect a high degree of phase synchronization and this will show up as a good correlation between the synchronization index and the magnetic field spatial plots. During the low-level contraction activity and also during the relaxation periods, uterine magnetic fields could still have a good amount of phase synchronization. This could be one of the main reasons for the observed differences in the corresponding windows of figures 9 and 10.

Magnetomyography offers a new, noninvasive technique for the evaluation of uterine signals. The synchronization index could be an indicator to track the spatial patterns of spread of uterine myometrial activity and thereby increasing our understanding of uterine contractions. In future studies, this application could contribute significantly to the development of methodologies for differentiating true and false labor and prediction of labor thus resulting in better management of pregnancies.
